# Relationship between Microsatellite Instability, Immune Cells Infiltration, and Expression of Immune Checkpoint Molecules in Ovarian Carcinoma: Immunotherapeutic Strategies for the Future

**DOI:** 10.3390/ijms20205129

**Published:** 2019-10-16

**Authors:** Hitomi Yamashita, Kentaro Nakayama, Masako Ishikawa, Tomoka Ishibashi, Kohei Nakamura, Kiyoka Sawada, Yuki Yoshimura, Nagisa Tatsumi, Sonomi Kurose, Toshiko Minamoto, Kouji Iida, Sultana Razia, Noriyoshi Ishikawa, Satoru Kyo

**Affiliations:** 1 Department of Obstetrics and Gynecology, Shimane University School of Medicine, Izumo 6938501, Japan; memedasudasu1103@gmail.com (H.Y.); m-ishi@med.shimane-u.ac.jp (M.I.); tomoka@med.shimane-u.ac.jp (T.I.); kohei320@med.shimane-u.ac.jp (K.N.); kiyoka-s@med.shimane-u.ac.jp (K.S.); y-yuki@med.shimane-u.ac.jp (Y.Y.); nagisa26@med.shimane-u.ac.jp (N.T.); kurose.s@med.shimane-u.ac.jp (S.K.); minamoto@med.shimane-u.ac.jp (T.M.); iida@med.shimane-u.ac.jp (K.I.); raeedahmed@yahoo.com (S.R.); satoruky@med.shimane-u.ac.jp (S.K.); 2 Department of Organ Pathology, Shimane University School of Medicine, Izumo 6938501, Japan; kanatomo@med.shimane-u.ac.jp

**Keywords:** microsatellite instability, ovarian cancer, immune checkpoint inhibitor, immunohistochemistry, mismatch repair protein

## Abstract

Ovarian cancer has the worst prognosis among gynecological cancers. Thus, new ovarian cancer treatment strategies are needed. Currently, immune checkpoint inhibitors such as anti-PD-1/PD-L1 antibody are attracting attention worldwide. The Food and Drug Administration approved the use of the PD-1 antibody pembrolizumab for solid cancers with microsatellite instability (MSI)-H or mismatch repair (MMR) deficiency in 2017. However, few studies on ovarian carcinoma have evaluated the relationship among MSI status, lymphocyte infiltration into the tumor, and the expression of immune checkpoint molecules by histologic type. We evaluated the expression of MMR proteins, tumor-infiltrating lymphocytes (CD8+), and immune checkpoint molecules (PD-L1/PD-1) by immunohistochemistry in 136 ovarian cancer patients (76, 13, 23, and 24 cases were high-grade serous, mucinous, endometrioid, and clear cell carcinoma, respectively) to investigate the effectiveness of immune checkpoint inhibitors. Only six cases (4.4%) had loss of MMR protein expression. There was no significant relationship between MSI status and age (*p* = 0.496), FIGO stage (*p* = 0.357), initial treatment (primary debulking surgery [PDS] or neoadjuvant chemotherapy) (*p* = 0.419), residual tumor after PDS or interval debulking surgery (*p* = 0.202), and expression of CD8 (*p* = 0.126), PD-L1 (*p* = 0.432), and PD-1 (*p* = 0.653). These results suggest that only a small number of MSI cases in ovarian cancer can be effectively treated with immune checkpoint inhibitor monotherapy. Therefore, to improve the prognosis of ovarian carcinoma, a combination therapy of immune checkpoint inhibitors and other anticancer drugs is necessary.

## 1. Introduction

Surgery, chemotherapy, and radiation therapy have been the mainstays of cancer treatments. However, advanced cancer and recurrent cancer have a poor prognosis with conventional treatments. Therefore, new therapeutic strategies are being sought. In recent years, immunotherapy has attracted attention worldwide. In general, immune cells recognize and attack cancer cells to suppress their growth. However, cancer cells have immune escape mechanisms, such as the programmed cell death-1 (PD-1)/PD-1 ligand (PD-L1) pathway [1,2]. The anti-tumor immune response mediated by cytotoxic T lymphocytes (CTLs) is suppressed when PD-1 expressed on CTLs binds to PD-L1 expressed on cancer cells. Immune checkpoint inhibitors that inhibit the PD-1/PD-L1 pathway have been clinically applied in various cancer types. However, the response rate of anti-PD-1 antibody was 20–30% in various cancer types; moreover, immune checkpoint inhibitors had serious adverse effects [3,4]. Therefore, identifying biomarkers for immune checkpoint inhibitors is important. The Food and Drug Administration approved the use of the anti-PD-1 antibody pembrolizumab for solid cancers with microsatellite instability (MSI)-H or mismatch repair (MMR) deficiency in May 2017 [5]. Tumors with many genetic mutations or mutation burden rich tumors are recognized as non-self, and thus, immune cells can infiltrate these tumors. Therefore, immune checkpoint inhibitors are considered to be effective in solid cancers with MSI-H and MMR deficiency [6,7].

The number of deaths due to ovarian cancer is increasing, and it has the worst prognosis among gynecological cancers [8]. Ovarian cancer is characterized by poor initial symptoms at the time of morbidity because the ovaries are located in the pelvis. Although the stage is an important prognostic factor in ovarian cancer, about half of the patients with ovarian cancer are identified in an advanced stage [9]. The mortality reduction effect of ovarian cancer screening methods such as transvaginal ultrasonography and measurement of CA125 has not been determined [10]. Currently, there is no effective screening method for ovarian cancer. Therefore, the discovery of an effective treatment for advanced ovarian cancer is necessary to improve prognosis.

According to the WHO classification in 2014, ovarian tumors are mainly divided into epithelial, sex cord-stromal, and germ cell tumors. Ovarian cancer is mostly epithelial, and representative histological types of epithelial ovarian cancer are serous, mucinous, endometrioid, and clear cell carcinoma [11].

Serous carcinoma is divided into high- and low-grade serous carcinoma, with most cases being high-grade serous carcinoma. *P53* mutations are frequently found in high-grade serous carcinoma, and many cases of high-grade serous carcinoma originate from the serous tubal intraepithelial carcinoma of the fallopian tube [12,13]. Endometrioid carcinoma and clear cell carcinoma are caused by endometriosis or endometriosis-related ovarian neoplasms, and these often have *ARID1A* and *PIK3CA* mutations [13,14,15,16]. Mucinous carcinoma originates from the mucinous cystadenoma (adenoma-carcinoma sequence) [13]. The sensitivity to chemotherapy depends on the tissue type of ovarian cancer. Although high-grade serous carcinoma is highly sensitive to anticancer drugs, mucinous carcinoma and clear cell carcinoma are resistant [17,18]. Although ovarian cancer is currently treated uniformly regardless of tissue type, it has different molecular and biological characteristics depending on the histologic type. Therefore, ovarian cancer should be treated according to the tissue type in the future.

The percentage of MSI in ovarian cancer was reported to be 2–20% [19,20,21,22,23]. Clear cell carcinoma and endometrioid carcinoma were reported to have many cases of MSI [19,23,24,25]. Few reports have evaluated the relationship among MSI status, lymphocyte infiltration into the tumor, and the expression of immune checkpoint molecules by histologic type in epithelial ovarian cancer.

This study aimed to investigate selected potential biomarkers of response to immunotherapy in ovarian cancer.

## 2. Results

### 2.1. Clinicopathological Factors

High-grade serous carcinoma, mucinous carcinoma, endometrioid carcinoma, and clear cell carcinoma was diagnosed in 76, 13, 23, and 24 cases, respectively. FIGO stage I/II was identified in 48 cases (serous 9 cases, mucinous 10 cases, endometrioid 11 cases, and clear 18 cases). FIGO stage III/IV was identified in 88 cases (serous 67 cases, mucinous 3 cases, endometrioid 12 cases, and clear 6 cases). Initial treatment performed was primary debulking surgery (PDS) and interval debulking surgery (IDS) after neoadjuvant chemotherapy (NAC) in 118 and 18 patients, respectively. Finally, 57 cases were R0 (no residual tumor) after PDS or IDS. TC therapy (paclitaxel 175 mg/m^2^ and carboplatin area under the curve = 5 mg/m^2^ every 3 weeks) or dose-dense TC therapy (paclitaxel 80 mg/m^2^ on day 1, 8, and 15 and carboplatin area under the curve = 5 mg/m^2^) were performed for NAC or postoperative adjuvant chemotherapy. Since 2013, patients with FIGO stage III/IV have been treated by including bevacizumab in the postoperative adjuvant therapy. Clinicopathological factors of patients are shown in Table 1.

### 2.2. Microsatellite Instability

By IHC, six cases (4.4%) (2, 1, 2, and 1 in serous, mucinous, endometrioid, and clear cell carcinoma, respectively) were identified as MSI. According to the chi-squared test, there was no significant relationship between MSI status and age (*p* = 0.496), FIGO stage (*p* = 0.357), initial treatment (PDS or NAC) (*p* = 0.419), and residual tumor (*p* = 0.202) (Table 1).

### 2.3. Relationship between MSI and Expression of CD8, PD-L1, and PD-1

According to the chi-squared test, there was no significant relationship between MSI status and expression of CD8 (*p* = 0.126), PD-L1 (*p* = 0.432), and PD-1 (*p* = 0.653) (Table 2, Table 3 and Table 4). Moreover, there was no correlation between MSI and the expression of CD8, PD-L1, and PD-1 in any histological type (high-grade serous, mucinous, endometrioid, and clear cell carcinoma).

**Figure 1:**
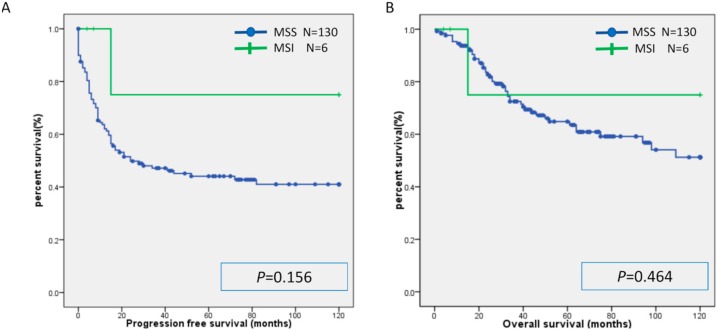
Kaplan-Meier analysis of progression-free survival (**A**) and overall (**B**) survival between the MSI group and MSS group. MSI: microsatellite instability; MSS: microsatellite stable.

**Figure 2:**
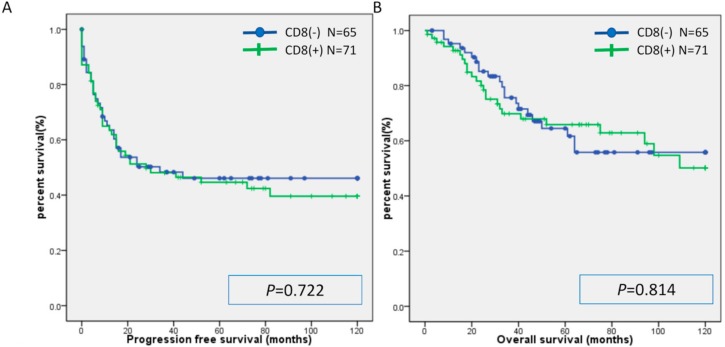
Kaplan-Meier analysis of progression-free survival (**A**) and overall (**B**) survival between the CD8 (+) group and CD8(-) group.

**Figure 3:**
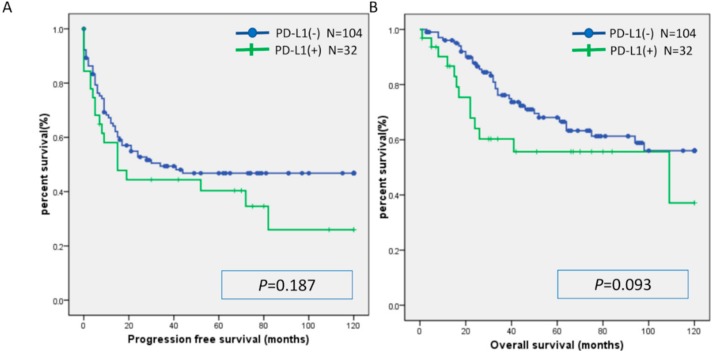
Kaplan-Meier analysis of progression-free survival (**A**) and overall (**B**) survival between the PD-L1(+) group and PD-L1(−) group.

**Figure 4:**
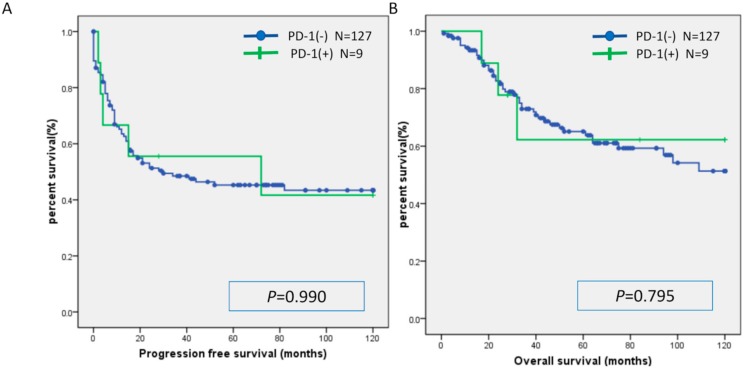
Kaplan-Meier analysis of progression-free survival (**A**) and overall (**B**) survival between the PD-1(+) group and PD-1(−) group.

There was no significant difference in PFS or OS between the MSI group and the MSS group (Figure 1). Similarly, there was no significant difference in PFS or OS between the CD8(+) and CD8(−) cases, PD-L1(+) and PD-L1(−) cases, and PD-1(+) and PD-1(−) cases (Figure 2, Figure 3 and Figure 4). Furthermore, there was no significant difference in PFS or OS by analysis combining CD8 and PD-L1 (Supplementary Figure S1).

### 2.4. MSI Analysis

**Figure 5:**
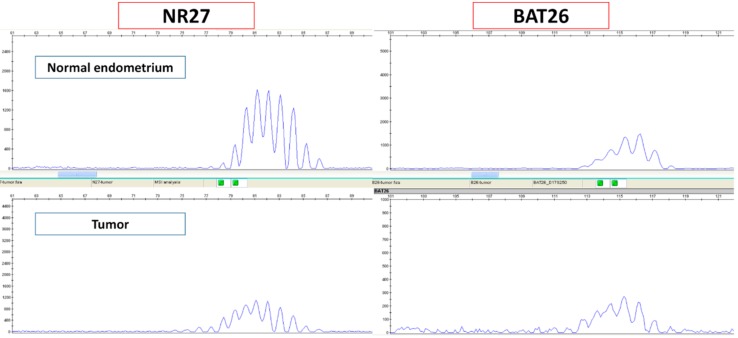
Cases that were evaluated as MSI by immunohistochemistry were further validated by MSI analysis.

Based on the MSI analysis, all six cases evaluated as MSI by immunostaining were confirmed to be MSI. (Figure 5).

## 3. Discussion

Ovarian cancer has the worst prognosis among all gynecological cancers, and the five-year survival rate is still below 50% in most countries. Survival trends between 1995–1999 and 2010–2014 were rather flat. The survival rate of ovarian cancer has not improved [8]. Therefore, new ovarian cancer treatment strategies are needed. Currently, numerous clinical trials are being conducted on the efficacy of immune checkpoint inhibitors in ovarian cancer [26]. Recently, the use of the PD-1 antibody pembrolizumab for solid cancers with microsatellite instability (MSI)-H or mismatch repair (MMR) deficiency was approved. Among gynecological cancers, the proportion of MSI cases in endometrial cancer was reported to be 15–30% [27,28]. We previously reported that MSI could be a biomarker for immune checkpoint inhibitors in endometrial cancer. The presence of tumor-infiltrating lymphocytes (CD8+) and PD-L1/PD-1 expression were significantly higher in the MSI group than in the MSS group in endometrial cancer [29]. Conversely, the proportion of MSI cases in ovarian cancer was reported to be 2–20%, which was less than that in endometrial cancer [19,20,21,22,23]. In the present study, only six (4.4%) patients were MSI-positive. Since among ovarian cancers, endometrioid carcinoma and clear cell carcinoma were reported to have a high proportion of MSI cases, immune checkpoint inhibitors might be effective in these carcinomas. Therefore, we evaluated CD8 lymphocyte infiltration into the tumor and expression of immune checkpoint molecules by IHC in each histological type of ovarian cancer. In this study, 2.6%, 7.7%, 8.7%, and 4.2% of high-grade serous carcinoma, mucinous carcinoma, endometrioid carcinoma, and clear cell carcinoma, respectively, were MSI cases. As reported previously, there were few cases of MSI in serous carcinoma [23]. MSI is caused by mutations in the mismatch repair genes (*MLH1*, *MSH2*, *MSH6*, and *PMS2*) or by methylation of the *MLH1* promoter. The presence or absence of *MLH1* methylation is currently being confirmed in the case of MLH1 loss by immunostaining. We plan to evaluate the mutation burden in MSI by using next generation sequencing. In this study, there was no correlation between MSI status and tumor-infiltrating lymphocytes (CD8+) and PD-L1/PD-1 expression. One study reports that the expression of CD8 and PD-1 in the MSI group was significantly higher than that in the MSS group in clear cell carcinoma [24]. In contrast, in case of endometrioid carcinoma, there was no correlation between MSI status and lymphocyte infiltration into the tumor [25]. Moreover, there was no correlation between MSI status and expression of CD8+, PD-L1, and PD-1 by histologic type. This may be due to the small number of MSI cases in ovarian cancer. These results suggest that immune checkpoint inhibitor monotherapy will be effective in only a few cases of ovarian cancer. The overall response rate of immune checkpoint inhibitor monotherapy in ovarian cancer was reported to be 6–22% [26]. There is currently no report on the relationship between the MSI status and effectiveness of immune checkpoint inhibitors in ovarian cancer in a clinical setting.

It has recently been reported that PD-L1 expression is upregulated by cancer treatment. Poly (ADP-ribose) polymerase inhibitor (PARP inhibitor) is used for initial maintenance therapy of platinum-sensitive BRCA mutation ovarian cancer or platinum-sensitive recurrent ovarian cancer. Ovarian cancer with homologous recombination deficiency (HRD) is sensitive to platinum drugs and PARP inhibitors [30,31,32,33]. Ovarian cancer with normal homologous recombination repair mechanism is considered to be less sensitive to PARP inhibitors and platinum drugs than ovarian cancer with HRD. When ovarian cancer with normal homologous recombination repair mechanism is treated with PARP inhibitors or platinum drugs, both the pathway of immune activation (neoantigen production due to repair errors and Interferon (IFN) response) and the pathway of immunosuppression through elevated PD-L1 expression are functional due to the normal homologous recombination repair mechanism [34]. Therefore, ovarian cancer with normal homologous recombination repair mechanism can be effectively treated with immune checkpoint inhibitors combined with PARP inhibitors or platinum drugs. It was reported that PARP inhibitors upregulated PD-L1 expression in breast cancer cell lines and mouse models. The combination of PARP inhibitor and anti-PD-L1 therapy had a significantly higher therapeutic efficacy than each agent alone [35].

Like PARP inhibitors, chemotherapy upregulates PD-L1 expression, suggesting that a combination of chemotherapy and immune checkpoint inhibitors may be more effective than chemotherapy alone [36,37]. In this study, there was no significant difference in PD-L1 expression between the PDS group and the NAC group (*p* = 0.578).

Programmed Cell Death 1 Ligand 2 (PD-L2), which is expressed on the surface of cancer cells, has recently attracted attention. It was reported that immune checkpoint inhibitors have a greater therapeutic effect in patients with high PD-L1 expression on tumor cells than in patients with low PD-L1 expression [38,39,40]. However, some PD-L1-positive patients respond poorly to anti-PD-1 antibodies [41]. Moreover, it was reported that anti-PD-1 antibody is effective even in patients without PD-L1 expression [41]. These results suggest that the molecular interactions with PD-1 including PD-L2 but not PD-L1 may predict the clinical response to immune checkpoint inhibitors. PD-L2 preferentially binds to PD-1 in competition with PD-L1 [41]. PD-L2 expression acts as a poor prognostic factor [42]. The relationship between the expression of PD-L1 and PD-L2 and the effect of anti-PD-1 antibody was investigated, and it was found that the response was greater in patients positive for both PD-L1 and PD-L2 than in those positive for only PD-L1 [43]. PD-L2 may be a biomarker for immune checkpoint inhibitors. Additionally, we will investigate the correlation between PD-L2 expression, immune cells infiltration, and expression of immune checkpoint molecules.

Furthermore, the regulatory T cell is an important factor in the tumor microenvironment. Regulatory T cells that infiltrate tumors express cytotoxic T-lymphocyte associated antigen-4 (CTLA-4), PD-1, and chemokine receptor 4 (CCR4) and suppress antitumor immune responses. In several carcinomas, the presence of regulatory T cells in tumor tissues was reported to be a poor prognostic factor [44]. It is believed that suppressing regulatory T cells is important to enhance the effects of immune checkpoint inhibitors. Activated regulatory T cells express CCR4. When the chemokine (CCL22), which is a ligand for CCR4, is produced in tumor tissues, regulatory T cells accumulate in tumor tissues. The CCR4 antibody can selectively suppress infiltration of tumor tissues by regulatory T cells. It was reported that the CCR4 antibody increases CD8+ T cells infiltration into tumors [45]. Clinical trials for combination therapy of anti-CCR4 antibody and immune checkpoint inhibitors are ongoing.

In addition, it was reported that the human leukocyte antigen class I (HLA class I) genotype influences the efficacy of immune checkpoint inhibitors. HLA- I molecules present neoantigens and virus particles. The antitumor activity of immune checkpoint inhibitors depends on HLA class I-dependent immune activity and CD8+ T cells. Data of the HLA- I genotype in advanced cancer patients treated with anti-PD-1 antibody or anti-CTLA-4 antibody was analyzed. Patients with the HLA-B44 supertype or maximal heterozygosity at HLA- I had extended survival. Contrarily, HLA-B62 supertype, homozygous for at least one HLA locus, or somatic loss of heterozygosity at HLA- I was associated with poor prognosis [46]. Based on these results, the genotype analysis of HLA may be important in determining the indication of immune checkpoint inhibitors.

In summary, there are a small number of MSI cases in ovarian cancer. Therefore, immune checkpoint inhibitor monotherapy will be effective only in these few cases. Combination therapy, including immune checkpoint inhibitors, may improve the prognosis of ovarian cancer. We are currently investigating the effectiveness of multi-drug therapy including immune checkpoint inhibitors in mouse models using ovarian cancer cell lines. Currently, several clinical trials are being conducted on the efficacy of immune checkpoint inhibitors in ovarian cancer. In addition, clinical trials on combination therapy including anticancer drugs and immune checkpoint inhibitors or molecular targeted therapeutic drugs and immune checkpoint inhibitors are being conducted [26]. Results from these clinical trials may open the door to new therapies for ovarian cancer.

## 4. Materials and Methods

### 4.1. Ethics Statement

This study was conducted according to the ethical standards of national and international guidelines as well as the Declaration of Helsinki and was approved by the institutional review board (Shimane University Hospital). Tumor specimens were collected after obtaining written consent from all patients with the approval of the Facility Ethical Committee (Shimane University Hospital, Izumo, Japan; approval No. 2004–0381, 5 March 2007).

### 4.2. Tissue Samples

We collected the tissue samples of ovarian carcinoma from 136 patients (76, 13, 23, and 24 patients with high-grade serous, mucinous, endometrioid, and clear cell carcinoma, respectively) treated between January 1997 and December 2017 in the Department of Obstetrics and Gynecology at the Shimane University Hospital. The samples were formalin-fixed and converted to paraffin-embedded tissue blocks. The samples were stained by hematoxylin and eosin and diagnosed by pathologists.

We adopted the guidelines of the International Federation of Gynecology and Obstetrics (FIGO) 2014 in the staging of surgery for ovarian cancer [47]. Histological diagnosis of ovarian carcinomas was performed according to WHO classification of tumors of the ovary 2014 [11].

The relevant clinical data were collected by retrospective review of the patient files. The follow-up period ranged from 1 month to 120 months, with a median follow-up of 42 months.

### 4.3. Immunohistochemistry

We evaluated the expression of MMR proteins (MLH1, MSH2, MSH6, and PMS2), immune checkpoint molecules (PD-L1 and PD-1), and CD8 infiltrations into the tumor by immunohistochemistry (IHC).

Formalin-fixed and paraffin-embedded sections (4 µm thick) were immunostained as described previously [29]. We used antibodies against MutL Protein Homolog 1 (1:50; Dako, Santa Clara, United States), MutS Protein Homolog 2 (1:50; Dako), MutS Protein Homolog 6 (1:50; Dako), Postmeiotic Segregation Increased 2 (1:40; Dako), PD-L1 (PD-L1 (SP263) Rabbit Monoclonal Primary Antibody; Roche, Basel, Switzerland), PD-1 (NAT105 Mouse Monoclonal Antibody; Roche), and CD8 (SP57 Rabbit Monoclonal Primary Antibody; Roche). Two researchers who were blinded to the clinical data evaluated the immunostaining samples by a light microscope.

Cases were evaluated as MSI when at least one of the four MMR proteins (MSH2, MSH6, PMS2, and MLH1) was negative. Other cases were evaluated as microsatellite stable (MSS). Expression of CD8 lymphocytes infiltrating the tumor was evaluated according to four levels (0, undetectable; 1+, low density; 2+, moderate density; and 3+, high density). Cases that were stained 2+ or 3+ were evaluated as positive. Cases were evaluated as positive for PD-L1 when ≥5% of the tumor cells were stained (membranous and cytoplasmic staining). Cases were evaluated as positive for PD-1 when ≥5% of the tumor-infiltrating lymphocytes were stained.

### 4.4. Microsatellite Instability Analysis

The six cases that were evaluated as MSI by IHC were further validated by MSI analysis. We extracted DNA and performed MSI analysis as described previously [27]. Eight microsatellite markers (BAT25, BAT26, D2S123, D5S346, D17S250, NR21, MONO27, and NR2) were used. If two or more markers showed length variation between the normal tissue samples (normal endometrium) and the tumor samples, we evaluated the cases to be MSI. If no length variation was found in any of the eight markers, we considered the cases to be MSS.

### 4.5. Statistical Analyses

We performed univariate analysis for progression-free survival (PFS) and overall survival (OS). PFS was defined as the period between the date of diagnosis and the date of first relapse. OS was defined as the period between the date of diagnosis and last follow-up. The data were represented as Kaplan-Meier curves. The log-rank test was used for statistical significance test. The chi-squared test was used to characterize the association between MSI and the expression of CD8, PD-1, and PD-L1. In the present study, we defined *p*-values below 0.05 as statistically significant.

## Figures and Tables

**Table 1:** Factors in patients with ovarian cancer.

Characteristic	MSI	MSS	*p*-Value
	*n* = 6	*n* = 130	
Age: number (%)			0.496
<60	3 (50)	54 (42)	
≥60	3 (50)	76 (58)	
FIGO Stage: number (%)			0.357
I, II	3 (50)	45 (35)	
III, IV	3 (50)	85 (65)	
Initial treatment (%)			0.419
PDS	6 (100)	112 (86)	
NAC	0 (0)	18 (14)	
Residual tumor after PDS or IDS (%)			0.202
No residual tumor (R0)	4 (67)	53 (41)	
Yes	2 (33)	77 (59)	

MSI: microsatellite instability; MSS: microsatellite stable; FIGO: International Federation of Gynecology and Obstetrics; PDS: primary debulking surgery; NAC: neoadjuvant chemotherapy; IDS: interval debulking surgery.

**Table 2:** Relationship between MSI status and CD8 expression.

Parameter	MSI	MSS	*p*-Value
	*n* = 6	*n* = 130	
CD8: number (%)			0.126
Positive	5 (83)	66 (51)	
Negative	1 (17)	64 (49)	

MSI: microsatellite instability; MSS: microsatellite stable.

**Table 3:** Relationship between MSI status and PD-L1 expression.

Parameter	MSI	MSS	*p*-Value
	*n* = 6	*n* = 130	
PD-L1: number (%)			0.432
Positive	2 (33)	30 (23)	
Negative	4 (67)	100 (77)	

MSI: microsatellite instability; MSS: microsatellite stable.

**Table 4:** Relationship between MSI status and PD-1 expression.

Parameter	MSI	MSS	*p*-Value
	*n* = 6	*n* = 130	
PD-1: number (%)			0.653
Positive	0 (0)	9 (7)	
Negative	6 (100)	121 (93)	

MSI: microsatellite instability; MSS: microsatellite stable.

## Supplementary Materials

Supplementary materials can be found at https://www.mdpi.com/1422-0067/20/20/5129/s1.

## Abbreviations

FIGO	International Federation of Gynecology and Obstetrics
MSI	microsatellite instability
MMR	mismatch repair
OS	overall survival
PFS	progression-free survival
PD-1	programmed cell death-1
PD-L1	programmed cell death-ligand 1
PDS	primary debulking surgery
NAC	neoadjuvant chemotherapy
IDS	interval debulking surgery
