# Supplementary material for: Relationship between Microsatellite Instability, Immune Cells Infiltration, and Expression of Immune Checkpoint Molecules in Ovarian Carcinoma: Immunotherapeutic Strategies for the Future
Source: Int J Mol Sci. 2019 Oct 16;20(20):5129. doi: 10.3390/ijms20205129 (PMC6829575; doi:10.3390/ijms20205129)
Supplement: Supplementary file 1 [file ijms-20-05129-s001.zip › supplementary figure1/Supplementary Figure1 legend.docx]

Supplementary Figure1: A, B, Kaplan-Meier analysis of progression-free survival (A) and overall (B) survival between CD8(-)/PD-L1(-) group and CD8(+)/PD-L1(+) group.
